# Hypernephroma Presenting with Cutaneous Leukocytoclastic Vasculitis and Lupus Anticoagulant: Resolution after Nephrectomy

**DOI:** 10.1155/2012/108230

**Published:** 2012-08-07

**Authors:** Nigel P. Murray, Amparo Ruíz, Eduardo Reyes

**Affiliations:** ^1^Instituto de Bio-Oncología, Universidad Mayor, Avenida. Salvador 95, Oficina 513, Providencia, Santiago 7500710, Chile; ^2^Unidad de Células Tumorales, Facultad de Medicina, Universidad Mayor, Renato Sánchez 4369, Las Condes, Santiago 27550224, Chile; ^3^Sección de Hematología, Servicio de Medicina, Hospital de Carabineros de Chile, Simón Bolívar 2200, Núñoa, Santiago 7770199, Chile; ^4^Servicio de Anatomía-Patología, Hospital de Carabineros de Chile, Simón Bolívar 2200, Núñoa, Santiago 7770199, Chile; ^5^Servicio de Urología, Hospital de Carabineros de Chile, Simón Bolívar 2200, Núñoa, Santiago 7770199, Chile

## Abstract

Hypernephroma can present as a variety of paraneoplastic, nonmetastatic conditions, including vasculitis, and rarely a lupus-type anticoagulant. Nephrectomy leads to the resolution of the systemic complaints. Malignancy, in this case hypernephroma, can present as an immune-mediated paraneoplastic syndrome which resolves after removal of the underlying tumor.

## 1. Introduction

60 years ago, Conley and Hartman published 2 cases of the lupus anticoagulant in patients with SLE [1]. This inhibitor was then found to be associated with other autoimmune disorders and recurrent spontaneous abortions [2, 3]. It has been associated with hematological malignancies such as lymphoma and acute myeloid leukemia. It has also been reported in association with solid tumors and in 4 cases with hypernephroma [4, 5]. The association of leukocytoclastic vasculitis and hypernephroma has rarely been documented [6, 7], and the combination of the three to our knowledge has not been reported previously in the literature.

We present a case of leukocytoclastic vasculitis in the presence of a lupus-type anticoagulant during its study a hypernephroma was discovered. We revise the few cases similarly described and possible physiopathological mechanisms.

## 2. Case Report

A 60-year-old woman, previously in good health and without a personal or family history of autoimmune disorders, presented to the Emergency Services with a 7-hour history of a right-sided hemiparesis and an expressive dysphasia. Physical examination confirmed signs of cerebellar dysfunction, a right-sided hemiparesis, and expressive dysphasia, and her physical examination was otherwise normal. CT head scan revealed a right-sided cerebellar infarct, and laboratory tests revealed a hemoglobin of 15.0 gr/dL (reference range 12–17 gr/dL), TTPK 39 s (range 22–40 s), prothrombin time 12 s (range 12–15 s), VHS 23 mm/hr, and C-reactive protein 0.7 mg/dL (range 0-1 mg/dL). Because of an allergy to aspirin she was started on ticlopidine 250 mg/day.

**Figure 1:**
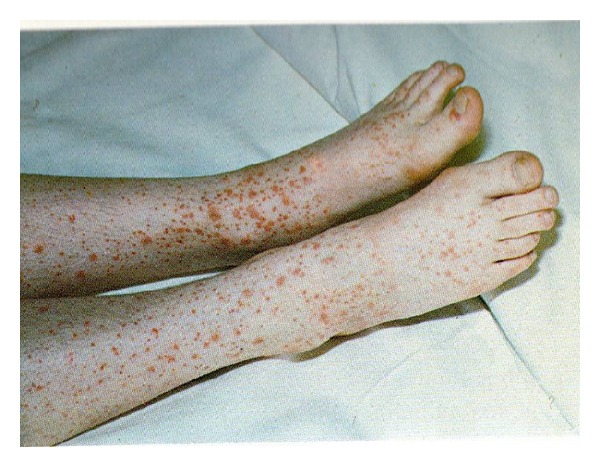
Vasculitis of the legs.

One month later the patient developed purpura, equimosis, peripheral edema, arthralgia, and hypertension arterial of 180/110. The lesions were localized to the feet, legs, and thighs, with hemorrhagic blisters around the ankles, some in the process of healing and were associated with pruritus (Figure 1). Further blood tests showed autoantibodies negative, crioglobulinemia negative, antihepatitis B and C negative, creatinine 1.06 mg/dL, hemoglobin 18.5 gr/dL, VHS 73 mm/hr, C-reactive protein 7.8 mg/dL, protrombina 13 s TTPK 56 s, and a diluted Russells Viper Venom test positive for a lupus-type inhibitor. Further questioning revealed three episodes of painless macroscopic hematuria during the previous month. 

The ticlopidine was discontinued and after skin biopsy, prednisone 60 mg/day was started for the vasculitis, as well as antihypertensive therapy. Because of the hematuria oral anticoagulation was not started. Skin biopsy revealed cutaneous detachment with necrosis of the epidermis; in the dermis there was a lymphocytic infiltration with leukocytoclasia, fibrinoid necrosis of the capillary walls, and extravasation of erythrocytes. Immunofluorescence showed deposits of C3*·* complement in the vascular endothelium of the middle and superficial dermis consistent with a leukocytoclastic vasculitis.

**Figure 2:**
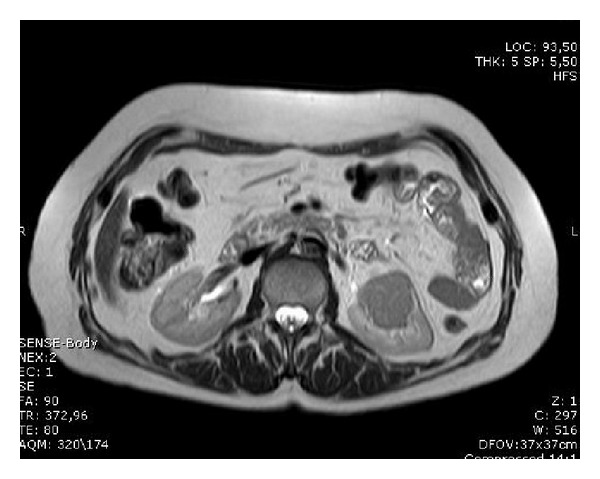
MRI abdominal showing hypernephroma of the left kidney.

**Figure 3:**
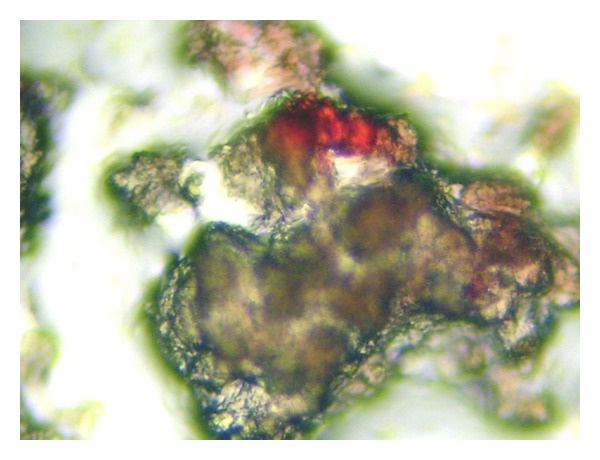
Hypernephroma cell (red) detected by immunocytochemistry in bone marrow.

A CT scan of thorax and abdomen revealed a 5 cm cystic tumor of the left kidney without evidence of metastasis (Figure 2). She underwent left radical nephrectomy without complications for a clear cell renal carcinoma grade II. There was no evidence of a vasculitis in the surgical specimen. Bone marrow biopsy showed positivity for hypernephroma cells, detected using immunocytochemistry with a monoclonal antibody antihypernephroma (DAKO), consistent with micrometastases (Figure 3).

Two months later, there was no clinical evidence of the vasculitis, the TTPK was normal, and tests were negative for anticardiolipins; the diluted Russell's Viper venom test was normal, as was the VHS, C-reactive protein, and hemoglobin. One year later, repeated bone marrow biopsy failed to show the presence of hypernephroma cells. She is without active treatment and is 6 years without evidence of recurrence. 

## 3. Discussion

The normal kidney produces erythropoietin, rennin, and angiotensin; therefore it is not surprising that in 1–5% of hypernephroma cases there is an erythrocytosis owing to overproduction of erythropoietin, or hypertension arterial secondary to overproduction of rennin and/or angiotensin. After surgery the patient returns to be normotensive as was seen in this case.

The lupus anticoagulant is a polyclonal immunoglobulin type IgG; it has been reported in autoimmune disease, monoclonal gammopathy of unknown significance, and AIDS. In all these diseases, there is a dysfunction of the immune system which suggests a common mechanism for the appearance of the lupus anticoagulant. Over the last few years there is accumulating evidence that in hypernephroma there is immune dysfunction; there is a decrease in the expression of transducer signaling proteins in T-lymphocytes and lack of maturity in the dendritic cells.

Experimental studies have shown that there is little or no recognition of renal tumor, cells by the immune system or activation of host defense mechanism by the tumor, and there is a complex immune dysfunction caused by the cancer [8]. There is defective antigen presentation by dendritic cells, partly related to the downregulation of costimulatory molecules such as B7.2 and decreased recruitment of CD8-positive cytotoxic lymphocytes [9]. Furthermore, there is production by the tumor of soluble immunosuppression mediators which decrease the T-lymphocyte response, increasing T-cell apoptosis [10], decreasing IL-2 production by B-lymphocytes CD4 helper cells [10] and inhibition of Jak-3 kinase activity produced by activation of the IL-2 receptor [11]. This may explain the increased incidence of autoimmune disease associated with hypernephroma.

Leukocytoclastic vasculitis is rarely associated with solid tumors, is more commonly found associated with infections such as EBV or HIV infection, endocarditis, hepatitis, and autoimmune disorders. The association with hypernephroma was first reported in 1976 by Andrasch et al. in a 63-year-old woman, since then there have been other cases reported in the literature [12]. In all the cases, the vasculitis was resolved after surgical removal of the tumor. 

The pathogenesis of this vasculitis is by immune complex deposition on the vascular wall and subsequent complement activation. This leads to the liberation of chemotactic substances, damaging the vascular wall, the decrease in fibrinolytic activity and leads to thrombosis. It is postulated that cytokine production by tumor cells is the trigger factor leading to autoimmunity [6, 7]. This may explain the increased incidence of autoimmune disease associated with hypernephroma [13]. What is important is that in all cases these paraneoplastic phenomenon disappeared with removal of the tumor, as was the case in this patient. While there is no tumor recurrence these features do not recur. 

## 4. Conclusions

In summary, we present a case of hypernephroma complicated by a paraneoplastic syndrome caused by immune-dysfunction, suggesting some of the possible mechanisms and that these clinical manifestations resolve once the tumor is removed. Hypernephroma causes multiple effects on the immune system, which can result in atypical presentaciones of the disease, as was the case in this patient.
